# Effects of kinesthetic haptic feedback on standing stability of young healthy subjects and stroke patients

**DOI:** 10.1186/s12984-015-0020-x

**Published:** 2015-03-13

**Authors:** Muhammad Raheel Afzal, Ha-Young Byun, Min-Kyun Oh, Jungwon Yoon

**Affiliations:** School of Mechanical & Aerospace Engineering & ReCAPT, Gyeongsang National University, Jinju, Republic of Korea; Department of Rehabilitation Medicine, Gyeongsang National University Hospital, Jinju, Republic of Korea

**Keywords:** Light touch, Kinesthetic haptic feedback, Stroke patients, Smartphone, Postural stability, Balance training

## Abstract

**Background:**

Haptic control is a useful therapeutic option in rehabilitation featuring virtual reality interaction. As with visual and vibrotactile biofeedback, kinesthetic haptic feedback may assist in postural control, and can achieve balance control. Kinesthetic haptic feedback in terms of body sway can be delivered via a commercially available haptic device and can enhance the balance stability of both young healthy subjects and stroke patients.

**Method:**

Our system features a waist-attached smartphone, software running on a computer (PC), and a dedicated Phantom Omni® device. Young healthy participants performed balance tasks after assumption of each of four distinct postures for 30 s (one foot on the ground; the Tandem Romberg stance; one foot on foam; and the Tandem Romberg stance on foam) with eyes closed. Patient eyes were not closed and assumption of the Romberg stance (only) was tested during a balance task 25 s in duration. An Android application running continuously on the smartphone sent mediolateral (ML) and anteroposterior (AP) tilt angles to a PC, which generated kinesthetic haptic feedback via Phantom Omni®. A total of 16 subjects, 8 of whom were young healthy and 8 of whom had suffered stroke, participated in the study.

**Results:**

Post-experiment data analysis was performed using MATLAB®. Mean Velocity Displacement (MVD), Planar Deviation (PD), Mediolateral Trajectory (MLT) and Anteroposterior Trajectory (APT) parameters were analyzed to measure reduction in body sway. Our kinesthetic haptic feedback system was effective to reduce postural sway in young healthy subjects regardless of posture and the condition of the substrate (the ground) and to improve MVD and PD in stroke patients who assumed the Romberg stance. Analysis of Variance (ANOVA) revealed that kinesthetic haptic feedback significantly reduced body sway in both categories of subjects.

**Conclusion:**

Kinesthetic haptic feedback can be implemented using a commercial haptic device and a smartphone. Intuitive balance cues were created using the handle of a haptic device, rendering the approach very simple yet efficient in practice. This novel form of biofeedback will be a useful rehabilitation tool improving the balance of stroke patients.

## Background

Humans use many natural sensors to maintain balance. These include the vestibular system, vision, proprioception (feedback from leg muscle movements), and tactile information from the soles of the feet. These cues are used to detect body tilt and to achieve balance [1,2]. The absence or inadequacy of any of these feedback systems reduces the cumulative input to the brain, which cannot now determine body orientation. The human body operates continuously in a self-balancing mode, minimizing body sway. An illness causing loss of one of the sensory inputs described above, or compromising bodily control, is problematic. Thus, stroke patients are prone to balance disorders causing difficulty in daily mobility and an increased risk of falls [3-6]. Therefore, both visual and auditory bio-feedbacks [7-14] have been used to reduce body sway in such patients and these procedures have been incorporated into balance training exercises [10-12]. The results [10-12] of task-oriented exercise program with altered sensory input could significantly improve standing balance by supplementing compromised sensory information during rehabilitation in order to retrain sensorimotor function [15], resulting in post-training improvements of postural stability.

Rehabilitation of balance can be also assisted by various haptic modalities. Light touch effectively reduces body sway [16]. Light touch refers to fingertip contact with another physical object. Sensorimotor information on body displacement afforded by contact of the index finger with a stationary bar can be used to stabilize balance and reduce body sway [16,17]. Light touch as a therapeutic mechanism can be a useful option in balance rehabilitation [18]. Fung et al. [19] examined the effect of a light touch cue on balance during standing and walking and found that a light touch of a finger along a fixed handrail improved postural stability in stroke patients walking on uneven surfaces. Boonsinsukh et al. [20] examined the effect of a light touch cue provided to post-stroke subjects via a cane on mediolateral (ML) pelvic stability during walking and found that this improved both muscle activity in paretic lower limbs and ML stability. In a recent study by Fung et al. [21], the use of virtual reality (VR), combined with manipulation of the physical environment and employment of a haptic cue, effectively enhanced post-stroke balance and mobility. A light touch improved postural stability during quiet standing, with or without somatosensory input from the fingertip, and this effect was eliminated when the hand was anaesthetized via placement of a compression block on the upper arm [22]. In a recent study, Albertsen et al. [23] showed that a light handgrip on a stick aided postural stabilization; the light grip facilitated delivery of a haptic cue under natural circumstances. Thus, recent studies have used light touch based haptic cue to improve postural stability in, and rehabilitation of, stroke patients. For active haptic cues, vibrotactile bio-feedback was found to be effective in reducing body sway during standing balance [15] and locomotion activities [24] in patients with vestibular deficit. Vibrotactile bio-feedback can provide haptic cue through vibration feedback to different parts of the body.

Haptic devices are widely used in virtual graphics environments to afford limited perception of mechanical properties such as force, vibration, and friction. Kinesthetic haptic interface exerts controlled forces on the human body using a passive connection that constantly remains in contact with the limbs of the operator. Thus, such cues may be used not only to deliver light touches reducing body sway during standing or walking, but also in balance training featuring virtual reality, affording patients more therapeutic options and improving development of cognition. Thus, it is necessary to study effects of kinesthetic haptic cues on postural stability which can be used to implement a virtual surface of reference, so that a light grip of the haptic device’s handle can be provided.

In the present work, we developed a simple and efficient system which assists the users in reducing body sway using kinesthetic haptic feedback, employing simple equipment including a smartphone (to sense body sway), a computer, and a low-cost commercially available haptic device. The system generates intuitive balance cues delivered via light grips of the handle of a Phantom Omni® device. Thus, the system is very simple to use but efficiently reduces body sway. To the best of the authors’ knowledge, this is the first system to use a haptic device for enhancing the postural stability. The system can provide a platform for possible balance training with complete manipulation according to clinical requirements by utilizing concept of light touch. The objective of the study is to develop a new system for balance improvement and testify the hypothesis that providing kinesthetic haptic feedback will reduce body sway in young healthy subjects with different postures and ground conditions, and stroke patients while standing in the stationary position. Below, we describe our system, give details of our subjects and protocols, describe the parameters measured, and the results. A discussion follows.

## Methods

### System description

**Figure 1:**
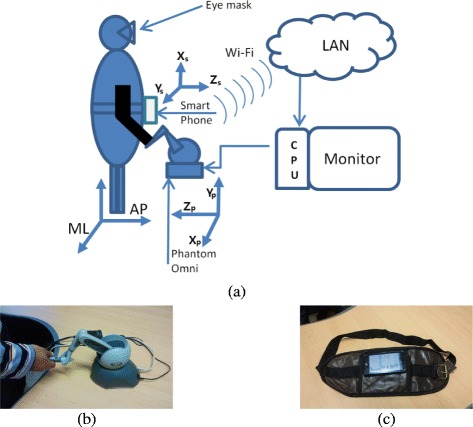
**The experimental setup. (a)** The system features a waist-attached smartphone, software running on a personal computer (PC), and a dedicated Phantom Omni® device for kinesthetic haptic feedback. **(b)** The Phantom Omni haptic device shown as used in the experiment. **(c)** The smartphone with its carrier waist belt.

The proposed kinesthetic haptic feedback system is composed of a smartphone, a computer, and a low-cost commercially available haptic device (a Phantom Omni® device). The proposed system featured a smartphone attached to the subject which measures the trunk tilt angles and sends the data through “Socket” program. The smartphone communicated via Wi-Fi and a wireless router. The PC is also connected to the router and shares the same network. The PC runs the “Socket” program and retrieves the data sent by the smartphone. The software running on the PC interprets the data received from the smartphone and generates the corresponding commands for Phantom Omni® device which then provides haptic feedback (Figure 1).

Smartphones have particular advantages [15] for the purpose of body sway measurement in terms of easily programmable and customizable body motion analyzers, and wireless communication. Previous researchers [14,15] have utilized smartphone as a reliable source of body tilt measurement device and they have developed Vibrotactile/Audio biofeedback based training systems for balance deficit patients. In our system the smartphone was physically attached to the waist with a leather belt, and sensed trunk tilt. An Android application ran continuously on the smartphone, recording ML and anteroposterior (AP) trunk tilt angles. The sagittal plane of the body was aligned with the *X*_*s*_*Z*_*s*_ plane of the phone, to allow measurement of AP tilt angles, and the frontal plane of the body was aligned with the *X*_*s*_*Y*_*s*_ plane of the phone to allow measurement of ML tilt angles. To calculate the raw tilt angles in smartphone, Android O.S standard development kit was used to access the sensory data from the smartphone at system. Using the Android SDK built in support for magnetometer and accelerometer, the rotation matrix for phone tilt angles was derived followed by calculation of orientation angles from the matrix. With use of smartphone application dedicatedly developed for our system objectives, we are able to achieve a sensing measurement resolution better than 0.1°. The data bandwidth of the smartphone utilized here was 10Hz, which was optimized to provide absolutely negligible system delay between sensory inputs of tilt from the smartphone to the input of the haptic device, including measurements on smartphone, transmission over Wi-Fi and decoding at the PC. For measuring significant body motion, medical studies tend to put the required sampling frequency much lower at 3-5Hz [25,26]. These trunk tilt data were sent to the router and the PC calculated changes in trunk tilt angles. The relevant software, written in Visual C++, continuously monitored the network card and decoded data packets from the smartphone to derive ML and AP tilt values.

The smartphone, an LG Optimus LTE LU6200, had a dual-core 1.5 GHz CPU and 1 GB of RAM, and efficiently ran the Android® 4.1.2. (Jelly Bean) software. Although the smartphone transmitted data continuously, these were not stored. Recording commenced when the operator manually pressed a key. The smartphone sent raw tilt angles but the program stored relative tilt angles, thus angles compared to initial ML and AP values. When a subject was ready, the operator pressed the button, and the program then loaded initial ML and AP values into buffers, and calculated changes in ML and AP values by comparing newly received values with these stored initial values. When the experiment finished, the operator pressed a key terminating data recording. The same program calculated the haptic forces delivered via the Phantom Omni® handle.

### Haptic feedback algorithm

The Phantom Omni® (position resolution 0.055 mm, peak force 3.3 N, and stiction 0.26 N) can be connected to and controlled by a PC [27]. The device is a well reputed haptic device which can produce directional force in the X_*p*_, *Y*_*p*_ and Z_*p*_ directions, having previously being featured in numerous research studies. Frequency response testing shows that Phantom Omni has higher than 40Hz bandwidth [28]. In our system, haptic force rendering was being performed at 1000 Hz and we provided a virtual reference surface by restraining the movement of the handle in the Y_*p*_ axis. The position of the reference surface was sustained by delivery of a force if a subject exerted any force in the Y_*p*_ axis of the haptic device. An output force from the haptic device was always less than 1 N and the handle was allowed to deviate if any subject exerted a force larger than 1 N. The ML and AP trunk tilt values were used to calculate the directions and magnitudes of all required forces; the handle then delivered these forces. The relationships between tilt angle and output haptic force magnitude and direction are given by eqns. (1) and (2):

1 $$ {F}_x= - k\kern0.5em .\kern0.5em \frac{trunk\ til{t}_{ML}}{range\ {X}_p} $$

2 $$ {F}_z=k\kern0.5em .\kern0.5em \frac{trunk\ til{t}_{AP}}{range\ {Z}_p} $$

where the “trunk tilt” is the tilt in ML or AP of the subject, calculated relative to the initial value as recorded at the start of the experiment, and the “range” in *X*_*p*_ and *Z*_*p*_ is the maximum permitted workspace (between −60 to +60 mm in both axis) of the haptic device. The stiffness “k” was set to 0.05 N/mm to reduce jerkiness, thus providing smooth force transfer and not affecting the body sway. Body sway was considered to be safe within the range of (positive and negative) 15 degrees with respect to the initial position in ML and AP. If the tilt was greater than this, the subject was considered to have lost control and haptic feedback would not be useful. Under such circumstances, the haptic device was constrained to stop providing feedback. This safe range of tilt was selected from pre-experiment trials since the maximum body sway exhibited by young healthy subjects was ranged between 10 to 15 degrees. A maximum latency of about 120 ms went oblivious to the users (the minimum system bandwidth of 8Hz), everything worked smoothly without disruption of measurement or haptic feedback and no reports of delayed feedback during experiment were made.

### Subjects

Young healthy subjects have previously been used to study the effects of the light touch in different conditions [16-18,22,23,29,30]. We therefore recruited such subjects to verify the effectiveness and observe the characteristics of our proposed system. We also performed trials for proof-of-concept with stroke patients in order to validate the use of our proposed system in increasing postural stability for the patients and demonstrate the possibility of implementing this novel form of biofeedback system to a balance training system. A total of 16 subjects, 8 healthy and 8 recovering from stroke, participated in the present study. Demographic details are given in Table 1.

**Table 1:** **Demographic data on subjects**

	**Subjects**	
	**Young healthy**	**Stroke patients**
**Participant no.**	8	8
**Male/Female**	6/2	6/2
**Age**	21–32 years	39–69 years
	(Mean = 26)	(Mean = 52)
	(SD = 3.3)	(SD = 11.9)
**Weight**	55–99 kg	56–66 kg
	(Mean = 75)	(Mean = 62)
	(SD = 14.7)	(SD = 5.8)
**Height**	158–185 cm	162–180 cm
	(Mean = 171)	(Mean = 169)
	(SD = 7.5)	(SD = 6.3)

No young healthy subject had any history of sensorimotor or neurological disorder. All stroke subjects were inpatients of the Rehabilitation Center of Gyeongsang National University Hospital (Jinju, Republic of Korea). Mini-Mental State Examination (MMSE) scores of stroke patients ranged from 15–30 with a mean value of 21.5 (30 Maximum). Two patients had bilateral hemiplegia, three left-side hemiplegia, and three had right-side hemiplegia. Mean duration after stroke onset was 70.0 ± 41.4 days (Mean ± Standard Deviation). Only one of the patients among the eight was suffering from infarction, rest of the seven suffered from hemorrhagic stroke. According to the six motor stages as defined by Brunnstrom [31], the eight patients who participated in this study had the following lower limb motor selectivity scores: V, V, IV, V, IV, V, V, V. All stroke patients had clear symptoms of lower and upper limb muscle deficiency on the paretic side and were unable to stand without support for more than 1 min. All subjects gave written informed consent in accordance with the rules of our local Ethics Committee.

### Protocol

The effect of kinesthetic haptic feedback on body sway was assessed in both healthy subjects and stroke patients. Different protocols were utilized for identifying the possible results from individual groups.

All subjects were required to stand still in front of a table, upon which the experimental apparatus including the Phantom Omni® was placed. Each subject faced the Phantom Omni® in a manner allowing the handle to be held with ease, without bending the body. The surrounding environment was designed to lack any stimulus. Subjects were instructed to remain silent and visual feedback was denied to young healthy subjects by having them done eye masks, which allowed the subjects to focus on the use of kinesthetic haptic feedback. All young healthy subjects tried to maintain balance while standing barefoot for 30 s. Young healthy subjects assumed each of four distinct postures, but patients only a single posture. Two of the four postures assumed by young healthy subjects involved standing on a highly unstable substrate, a high-density foam of dimensions 600 × 600 × 150 mm. This simulated soft ground. Task details are shown in Table 2. Kinesthetic haptic feedback was either provided or not. Thus, each young healthy subject underwent eight tests.

**Table 2:** **Postural conditions assumed by young healthy subjects**

**Posture**	**Conditions**
P1:	Standing with one foot on the ground and the head facing forward.
P2:	Standing heel-to-toe (the Tandem Romberg stance) with the head facing forward.
P3:	Standing on one foot on foam with the head facing forward.
P4:	Standing heel-to-toe on foam with the head facing forward.

**Figure 2:**
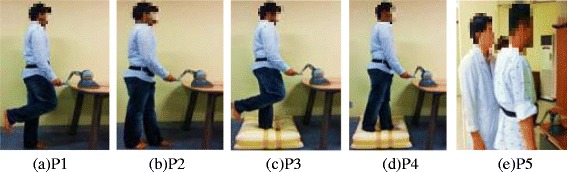
**Postures assumed by young healthy subjects and stroke patients.** Young healthy participants performed balance tasks with eyes closed after assumption of each of four distinct postures for 30 s; **(a)** one foot on the ground(P1); **(b)** the Tandem Romberg stance(P2); **(c)** one foot on foam(P3); **(d)** the Tandem Romberg stance on foam(P4); **(e)** Stroke patients performed balance task with eyes open and standing in Romberg stance (P5) for 25 s.

Stroke patients were infirm and assumed a different posture, P5. The lower limb muscles were weak and the patients lacked self-confidence. Thus, patients were not asked to cover their eyes and did not assume any complex posture. This experiment condition can be acceptable since balance training exercises are generally performed with eyes open conditions to ensure the safety of patients. Patients were instructed to focus their gaze on a plain wall in front of them to minimize visual effects and fully utilize the kinesthetic haptic feedback for balance aid. The chosen posture, P5, can be defined as use of the Romberg stance with the feet as close together as possible. All patients tried to maintain balance while barefoot for 25 s. Physical therapists and physicians specializing in rehabilitation were in attendance at all times and monitored the process. Figure 2 shows the above mentioned postures of the subjects.

Feedback/no feedback was randomly selected in all subjects. Thus, the first test of any type featured random selection between no feedback and feedback. Feedback was a light directional force indicating how the body should be moved to regain balance. Thus, the handle indicated that where the body should be tilted. Patients with one-sided hemiplegia were asked to use their non-paralyzed hands, whereas those with hemiplegia on both sides, and normal subjects, were asked to use their preferred hand. The experimental concept was explained to all subjects and they were told how a light touch of the Phantom Omni® device would provide assistance.

### Data analysis

All trunk tilt values of ML and AP values were stored in the computer and later analyzed using the MATLAB® software. Projection of trunk tilt (PT) was calculated from the data of trunk tilt angles and smartphone’s attachment height, given by eqns. (3) and (4):

3 $$ P{T}_{ML}= trunk\ til{t}_{ML}*h\kern4.75em \left(\mathrm{cm}\right) $$

4 $$ P{T}_{AP}= trunk\ til{t}_{AP}*h\kern4.75em \left(\mathrm{cm}\right) $$

here “*h*” is the height of smartphone’s attachment to the subject’s trunk from ground up. Since the tilt angles are small, PT can be linearized as eqns. (3) and (4). Similar to our approach, other researchers have used trunk tilt projection derived from an electromagnetic sensor, identified balance and stability behavior, and classified individuals on the basis of age, gender, height and weight [32-35]. Mean Velocity Displacement, Planar Deviation, and the ML and AP Trajectories [Eqns. (5)–(8) below] were also measured as parameters of body sway.

5 $$ MVD=\frac{{\displaystyle \sum}\frac{\sqrt{\left({\left({\mathrm{PT}}_{\mathrm{ML}}\left(\mathrm{i}\right)-{\mathrm{PT}}_{\mathrm{ML}}\left(\mathrm{i}-1\right)\right)}^2+{\left({\mathrm{PT}}_{\mathrm{AP}}\left(\mathrm{i}\right)-{\mathrm{PT}}_{\mathrm{AP}}\left(\mathrm{i}-1\right)\right)}^2\right)}}{{\mathrm{t}}_{\mathrm{i}}-{\mathrm{t}}_{\mathrm{i}-1}}}{\mathrm{n}}\kern4.75em \left(\mathrm{cm}/\mathrm{s}\right) $$

6 $$ PD=\sqrt{\upsigma^2{\mathrm{PT}}_{\mathrm{ML}}+{\upsigma}^2{\mathrm{PT}}_{\mathrm{AP}}}\kern4.75em \left(\mathrm{cm}\right) $$

7 $$ MLT={\displaystyle \sum}\left|{\mathrm{PT}}_{\mathrm{ML}}\left(\mathrm{i}+1\right)-{\mathrm{PT}}_{\mathrm{ML}}\left(\mathrm{i}\right)\right|\kern4.75em \left(\mathrm{cm}\right) $$

8 $$ \mathrm{APT}={\displaystyle \sum}\left|{\mathrm{PT}}_{\mathrm{AP}}\left(\mathrm{i}+1\right)-{\mathrm{PT}}_{\mathrm{AP}}\left(\mathrm{i}\right)\right|\kern4.75em \left(\mathrm{cm}\right) $$

MVD is the mean value of all PT velocities; changes in the ML and AP are combined to yield a single velocity value. PD is defined as the square root of sum of variances (*σ*^2^) of PT displacement in ML and AP directions. Variance of PT displacement measures show how far the PT is spread out. Similarly the sums of changes in ML and AP projection of tilt yield MLT and APT, respectively. Consequently, a larger value of the above mentioned parameters [Eqns. (5)–(8)] indicate the greater balance difficulty. Q-Q plot evaluation tool was utilized to observe the distribution of data, which was found to be lying within acceptable range of normal distribution. To assess the significance of body sway reduction upon provision of kinesthetic haptic feedback, a one-way ANOVA has been used.

## Results

**Figure 3:**
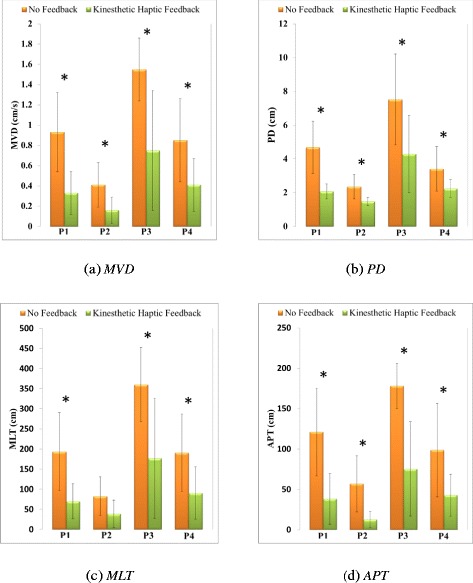
**Body sway of young healthy subjects assuming each of four postures and either afforded feedback or not.** Kinesthetic haptic feedback reduced body sway in young healthy subjects regardless of posture or ground condition. All index values exhibited significant (p < 0.05) reductions in body sway upon application of kinesthetic haptic feedback. Differences (feedback/no feedback) in the MLT data from subjects in posture P2 were of borderline significance (p = 0.055). ***(a)*** MVD ***(b)*** PD ***(c)*** MLT ***(d)*** APT. *p-value < 0.05.

**Figure 4:**
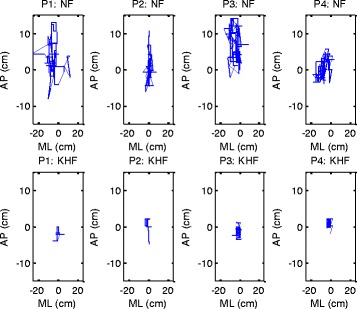
**Statokinesigram of one young healthy subject’s test:** In all four postures significant reduction in body sway is shown as a result of kinesthetic haptic feedback. NF: No Feedback, KHF: Kinesthetic Haptic Feedback.

The means, standard deviations and p-values (derived by comparative ANOVA) of data from young healthy subjects are shown in Table 3. Kinesthetic haptic feedback significantly reduced (p-values <0.05) the MVD, PD, MLT, and APT parameters of body sway when any of the four postures was assumed. Figure 3 compares the extents of body sway with and without feedback. Significant reduction in the statokinesigram of one young healthy subject’s test is shown in Figure 4.

**Table 3:** **Body sway parameters of young healthy subjects**

**Analysis parameter**	**Posture**	**No Feedback (NF)**		**Kinesthetic Haptic Feedback (KHF)**		**Comparison of NF and KHF**
		**Mean**	**SD**	**Mean**	**SD**	**p-value**
MVD (cm/s)	P1	0.93	0.39	0.33	0.21	0.002
	P2	0.41	0.22	0.16	0.13	0.019
	P3	1.55	0.31	0.75	0.59	0.004
	P4	0.85	0.41	0.41	0.26	0.022
PD (cm)	P1	4.68	1.55	2.07	0.44	0.001
	P2	2.35	0.73	1.48	0.25	0.006
	P3	7.52	2.43	4.29	2.29	0.016
	P4	3.41	1.32	2.24	0.52	0.036
MLT (cm)	P1	193.20	96.84	69.87	43.69	0.005
	P2	82.47	48.30	38.75	34.13	0.055
	P3	360.16	92.05	176.77	149.29	0.010
	P4	190.83	96.43	90.84	65.07	0.029
APT (cm)	P1	121.18	54.34	38.44	31.54	0.002
	P2	57.08	34.90	12.71	10.24	0.003
	P3	178.03	28.16	75.35	58.39	0.001
	P4	98.66	57.71	42.87	26.14	0.025

**Figure 5:**
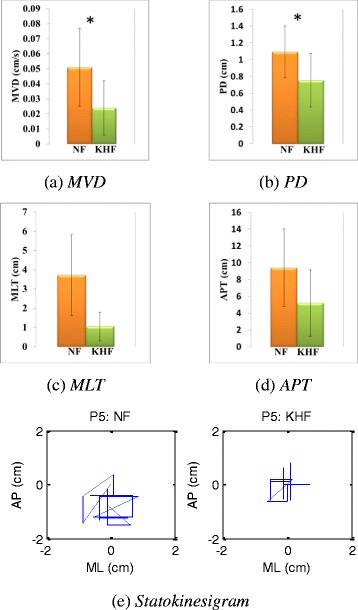
**Reduction in body sways of stroke patients receiving kinesthetic haptic feedback.** Stroke patients mustered the confidence to use the system, and all indices showed that feedback reduced sway. The p-values for the MVD and PD comparisons were significant (0.041 and 0.047 respectively). ***(a)*** MVD ***(b)*** PD ***(c)*** MLT ***(d)*** APT ***(e)*** statokinesigram of one single patient’s test. *p-value < 0.05. NF: No Feedback, KHF: Kinesthetic Haptic Feedback.

The results of tests performed on stroke patients assuming posture P5 are shown in Table 4. All parameters showed that the body sway of stroke patients decreased when feedback was provided, and the MVD and PD parameter exhibited significant values (p < 0.05 for both comparisons). Figure 5 compares the extent of body sway under no feedback and feedback conditions.

**Table 4:** **Body sway parameters of stroke patients**

**Analysis parameter**	**No Feedback (NF)**		**Kinesthetic Haptic Feedback (KHF)**		**Comparison of NF and KHF**
	**Mean**	**SD**	**Mean**	**SD**	**p-value**
MVD (cm/s)	0.051	0.026	0.024	0.018	0.034
PD (cm)	1.095	0.308	0.756	0.316	0.047
MLT (cm)	3.717	4.224	1.054	1.459	0.114
APT (cm)	9.421	4.626	5.209	3.950	0.070

## Discussion

Balance and postural control are defined as the ability to maintain the Center of Gravity (COG) over the base of support (BOS) within a given sensory environment, and are influenced by complex neuromuscular and skeletal processes [36]. Good balance and postural control are required for safe walking and performance of daily activities. Lack of such attributes leads to falls [37]. Both musculoskeletal and neurological factors including the vestibular system, vision, proprioception, muscle strength, and cognition are involved, in a complex manner, in postural and balance control [38]. Light touching of fingers or a hand can be used as an aid to balance, reducing body sway. Previously [16-19,21-23,29,30], light touches (controlled forces delivered through environmental surfaces) were confirmed to reduce body sway. In the present work, we developed a novel balance enhancement system delivering kinesthetic haptic feedback to the hand via the handle of a Phantom Omni® device. Young Healthy subjects and stroke patients were tested with eyes closed and open, respectively.

Any haptic feedback scheme should be carefully selected to enhance the stability of balance via kinesthetic haptic feedback. Wing et al. [39] used a haptic device to deliver a “light tight touch” to an index finger held in a thimble and increased the extent of AP sway via either entraining or by causing the device to emit either simple or complex AP sinusoidal oscillations. In preliminary work, Albertsen et al. [23] verified that a light touch (LT) and a light grip (LG) on a stable support yielded equivalent postural stabilization, and showed that resistance to stick movement facilitated postural stabilization. Our kinesthetic haptic scheme delivering feedback on trunk tilt angles worked well in the conducted trials.

### Effect of kinesthetic haptic feedback on young healthy subjects

Kinesthetic haptic feedback significantly reduced body sway in young healthy subjects. In the sagittal plane, the center of the body naturally lies in front of the ankle, and a tendency toward AP sway is evident. In the frontal plane, the bridge-like frame formed by the legs and pelvis, with the center of the body in the middle, tends to minimize ML sway. The feet are placed heel-to-toe in the Tandem Romberg stance and the slightest sideways displacement in either direction causes sway in that direction [39]. The one-legged P1 posture creates both ML and AP instability. However the Tandem Romberg stance of posture P2 is likely to be associated with more ML sway. Postures P3 and P4 are simply postures P1 and P2 assumed on soft ground, decreasing subject self-confidence and favoring more sway. Feedback reduced body sway in young healthy subjects regardless of posture or ground condition (Table 3). MVD, PD, MLT and APT are the imperative body sway assessment parameters as discussed earlier in the section of data analysis. In all of these parameters, small numerical values express reduction in body sway. From Table 3, it is understandable that all parameter values exhibited significant reductions in body sway upon application of kinesthetic haptic feedback. Differences (feedback/no feedback) in the MLT data from subjects in posture P2 were of borderline significance (p = 0.055). The sway reduction by kinesthetic haptic feedback in P1 is significant as it was observed when light touch was utilized [16]. Likewise, results of reduced body sway in P2 by kinesthetic haptic feedback and by utilization of light touch [17] are both significant. With the kinesthetic feedback, one leg standing postures (P1and P3) exhibited more improvements than tandem Romberg postures (P2 and P4), complying the usage of the feedback method proposed better for more balance demanding postures. Lesser body sway was observed in standing on ground postures (P1and P2) than standing on foam postures (P3 and P4); this particular effect of foam on increased postural sway has been observed in previous studies [30] about light touch. The reduction in body sway assessment parameters on application of feedback was also more evident in standing on ground postures (P1and P2) than standing on foam postures (P3 and P4), implying that our system does not actively compensate for vertical direction disturbances caused by ground conditions. It was observed in all trials that AP sway was less than ML sway. Similar observations are found in the previous studies about light touch [30]. Trials performed on young healthy subjects verify that; kinesthetic haptic feedback works significantly well to reduce body sway, similar to the earlier researches [16,17,30] in which increase in postural stability was enhanced by light touch. These findings imply that kinesthetic haptic feedback provide analogous input as delivered by light touch.

### Effect of kinesthetic haptic feedback on stroke patients

Stroke patients often assume asymmetrical postures and experience walking difficulties caused by reduced muscular power, imbalanced weight distribution, impaired proprioception, an exaggerated stretch reflex, spasticity, and impaired motor control [40]. The risk of falls and other injuries increases. After a stroke, it is essential to restore adequate postural control. In our present study, we used a Phantom Omni® device to provide intuitive assistance reducing body sway. Stroke patients mustered the confidence to use the system, and all parameters showed that feedback reduced sway (Table 4). The p-values for the MVD and PD comparisons between no feedback and feedback conditions were significant (0.034 and 0.047 respectively). Both MVD and PD are important in balance assessment. Overall, sway was significantly reduced but differences in test and control MLT and APT values, which are heavily dependent on exact projection of trunk tilt displacements, did not attain significance. Mini Mental State Examination (MMSE) scores were not high for patients as the average score was 21.5. Two patients out of eight were low in absolute scores of MMSE as 15. However, this score may arise from low educational level of the patients, since the calculation and orientation score of the MMSE were low. All patients were able to communicate and conduct three step commands such as receiving paper, folding into half and then giving back to the examiner with normal comprehension, so it did not affect the study. While participating in the experiment, they followed the protocol instructions without any difficulty. The patients were able to feel and interpret the feedback effectively. In the post experiment data analysis Pearson correlation coefficients were calculated between the MMSE score of each patient and improved parameter values of body sway. Correlation of MMSE with MVD (0.24), with PD (0.46), with MLT (0.16) and with APT (0.22) was found to be not linear at all. These results clearly show that reduction in body sway occurred regardless of MMSE value and our kinesthetic haptic feedback system generated haptic cues which were intuitive and helpful for patients. Stroke patients were clearly motivated to stand and balance. Our system efficiently reduced the body sway of stroke patients who were suffering balance disorder. Thus, suggesting this new system is capable of providing a new therapeutic solution in balance rehabilitation.

### Possible effect of kinesthetic haptic feedback on balance rehabilitation

Many conventional treatments including neurodevelopmental training (NDT) and sensory stimulation have been used to help stroke patients shift weight to the affected side. In addition, many walk training methods including weight-unloading devices, robots, the Balance Master System, and the Balance Retrainer, have been developed to encourage patients to shift weight to the affected side [41-47]. The outcomes of such training remain controversial. Use of our kinesthetic haptic feedback system by stroke patients may improve their clinical parameters of balance. Factors such as limits of stability (LOS), Berg Balance scale (BBS) and Clinical Test of Sensory Interaction and Balance (CTSIB) are warranted to be observed after balance training on this system which will be presented in the future research. Further, the system may serve as a therapeutic option for treatment of central and peripheral diseases causing impairments in balance and gait. Such treatment would complement physical and pharmacological therapy. The major limitations of our study include a small sample size, a relatively slow update rate, simplified estimation of trunk tilt projection in upright posture which cannot include the possible effects of motion at hip, lack of measurement of changes in dynamic balance, and no long-term follow-up. Additional outcomes, including muscle strength, sensory parameters, features of gait, and ability to perform the activities of daily life, should be evaluated in future trials. Despite the several limitations mentioned above, we have shown that kinesthetic haptic feedback may complement traditional treatments that seek to improve posture and balance control. Moreover, the system presented in this study has high potentials to be setup in patients’ houses or outpatient clinics for balance training exercises. Other balance training systems like Biodex and Balance Master are not a comprehensive option for stroke patient’s in-house balance training due to high cost and complex operations.

## Conclusion & future work

We present a new and effective form of biofeedback reducing body sway via kinesthetic haptic feedback. The system uses a Phantom Omni® device and a smartphone, generating intuitive balance cues via light grips delivered by the handle of the haptic device. Our system is simple yet efficiently reduces body sway in both young healthy subjects and stroke patients. Our new tool may be used to rehabilitate standing balance in stroke patients, in conjunction with other techniques including visual and haptic multi-modal training, and haptic dynamic balance assistance during walking. In future, we will study multi-modal balance training schemes featuring both haptic and visual feedback and the haptic effects achievable during motion as well as dealing with cognition effects with haptic feedback during balancing. Clinical trials will address the effects of kinesthetic haptic feedback as an alternative to traditional balance training protocols.

## Abbreviations

PC: Personal Computer
ML: Mediolateral
AP: Anteroposterior
MVD: Mean velocity displacement
PD: Planar deviation
MLT: Mediolateral trajectory
APT: Anteroposterior trajectory
ANOVA: Analysis of variance
MMSE: Mini-mental state examination
SD: Standard deviation
NF: No feedback
KHF: Kinesthetic haptic feedback
PT: Projection of trunk tilt
